# Taffit: An Excel Tool for Fitting Tafel Data

**DOI:** 10.1021/acsmeasuresciau.5c00038

**Published:** 2025-07-15

**Authors:** Joshua Coduto, Johna Leddy

**Affiliations:** Department of Chemistry, 4083 University of Iowa, Iowa City, Iowa 52242, United States

**Keywords:** Tafel plot, Tafel slope, exchange current density, Taffit, HER on glassy carbon electrode, electrocatalysts, Tafel analysis

## Abstract

Tafel analysis is widely used to characterize electrode kinetics. The technique has found use in electrochemistry, catalysis, materials, and corrosion research. Accurate Tafel analysis is especially critical in comparison of electrocatalysts. However, classical Tafel analysis (CTA) relies on the user’s subjective selection of a linear range in the Tafel plot; dependent on linear regression of the user-selected range, kinetic parameters can vary by orders of magnitude. As use of CTA in the literature grows, a need is identified for more reliable, user-independent Tafel analysis. Here, Taffit, an algorithm constructed in the widely available Microsoft Excel, is presented. Taffit generates a Tafel plot from linear sweep voltammetric data and determines the exchange current density *j*_0_, charge transfer coefficient α, and Tafel slopes by closest statistical fit. Comparisons between Taffit and CTA are made for the hydrogen evolution reaction (HER, 2H^+^ + 2*e* ⇌ H_2_) on glassy carbon (GC) and platinum electrodes. Taffit finds log *j*_0_ values of −7.2 and −3.9 for GC and Pt under H_2_ at pH 0, as measured without resistive compensation. This is the first report of *j*_0_ for HER on GC. Because algorithmic fitting in the low overpotential region uses both cathodic and anodic branches of the Tafel plot, Taffit has greater precision than CTA. Agreement is also shown between literature values reported by CTA and those obtained by Taffit for HER on metal phosphide and selenide electrocatalysts. The Taffit algorithm substantially reduces subjectivity to improve the accuracy and precision of Tafel analysis.

## Graphical abstract

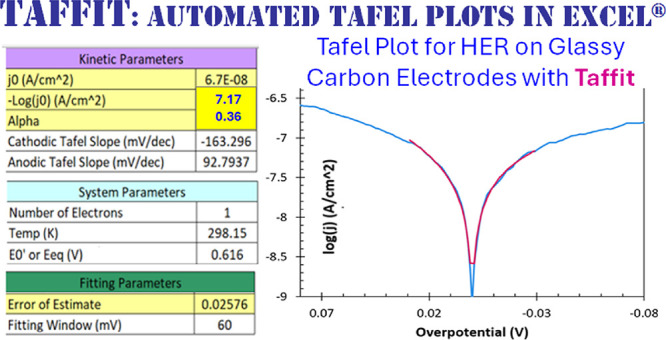

## Introduction

Tafel analysis is a technique to measure and characterize interfacial heterogeneous electron transfer rates. Used in many fields, including electrochemistry, catalysis, materials science, and corrosion, competent Tafel analysis is especially critical for comparing different electrocatalysts. Tafel analysis utilizes a log plot of current density, log *j* versus the electrode potential applied relative to the equilibrium potential, the overpotential η. At its simplest, a linear region is identified, and kinetic parameters are extracted from the slope (charge transfer coefficient α) and intercept (exchange current density *j*_0_) over the selected region. Extensions of Tafel theory model effects of surface coverage, multistep mechanisms, and competing reactions.^1–8^ Though advanced models and analysis tools are presented in the literature, many researchers employ what is here designated as classical Tafel analysis (CTA). This method is mathematically simple and approachable to scientists with varied backgrounds, but fundamentally and mathematically, CTA is subject to constraints that are not always recognized. Without careful attention to the data analysis, CTA can be compromised by user bias that erroneously overestimates electrocatalytic rates.

The challenges of well-deployed CTA are several. In part, CTA is limited by the required user identification of a linear region for the regression and analysis.^9–11^ CTA is also limited by fundamental theory and mathematical approximations.^12^

CTA is based on the Butler–Volmer equation for faradaic current measured without mass transport limitations. Faradaic current measures only interfacial electron transfer flux, independent of nonfaradaic charging currents and voltage drops due to uncompensated solution resistance. CTA is limited at more extreme overpotentials, where mass transport rates increase. As η approaches zero, CTA is limited by mathematical approximations needed to establish the linearized rate expression of CTA. The linearizations require that the current is set solely by either the oxidation or the reduction. CTA cannot be applied about the minimum log *j* where η → 0. Mass transport and only either the reduction or the oxidation limit the number of digitized data points available for valid CTA, especially at high electron transfer rates. In CTA, selection of the linear region is constrained by fundamental, mathematical, and digitization constraints. Furthermore, multiple values for kinetic parameters (e.g., *j*_0_, α, and Tafel slopes) can be found within regions deemed acceptable within the Butler–Volmer (BV) framework. More sophisticated constraints on CTA for determining Tafel slopes are reported.^11^ Constraints and variation in CTA lower measurement confidence and diminish the quality of rate parameters reported in the literature.

Alternative algorithmic methods reduce or eliminate human subjectivity in Tafel analysis to better determine kinetic parameters. Potentiostat platforms identify linear regions where CTA might be applied, but the platforms do not determine whether the data fall into the region where Tafel analysis is applicable. Other methods endeavor to model Tafel data directly by simulation or model of the Tafel data.^13–18^ A limited number of algorithmic tools for data analysis are available.^13–15^ These tools utilize various equations, methods, and data types. Notably, Agbo and Danilovic, created a general tool for extracting Tafel slopes by fit of the entire Tafel plot, including data that would otherwise fall into a range where Tafel analysis does not apply.^14^ These methods require knowledge of programming. While powerful, coding requirements present a barrier for researchers who do not have the requisite experience. Moreover, researchers continue to report CTA results in spite of available tools.^19–26^

This work presents a more accessible Tafel analysis tool, Taffit, for the automatic fitting of Tafel plots based on the underlying electron transfer theory. Taffit overcomes several limitations inherent to CTA. Taffit is built in the ubiquitous Microsoft Excel and is designed for use by both novices and experts in fundamental Tafel analysis. Taffit is designed as a replacement for CTA that eliminates subjective identification of a linear region. Like CTA, Taffit does not apply where mass transport impacts current. Unlike CTA and all Tafel analyses, Taffit applies simultaneously to both the anodic and cathodic branches of the Tafel plot. This increases the number of available data points and avoids the mathematical constraints of the CTA linearization. Taffit applies for concurrent oxidation and reduction currents. Statistics for nonlinear regression identify the fit to available data. Taffit provides the best statistical fit to log *j*_0_ versus η data; Taffit does not relieve the user from evaluating the significance of the reported parameters. To demonstrate Taffit, comparisons are provided between Taffit and CTA for both experimental and literature data.

### Classical Tafel Analysis

CTA is fundamentally an extension of BV kinetics.^12^ BV kinetics apply where faradaic current is not limited by mass transport. Consider an *n* electron transfer reaction between species O and R, where *z* is the charge.

$$O^{z}+ne\overset{k_{f}}{\underset{k_{b}}{⇄}}R^{z-n}$$ 1

The forward and backward rates of reaction are defined by rate constants *k*_f_ and *k*_b_. At an electrode, *k*_f_ and *k*_b_ (cm/s) are potential dependent. Current density *j* (A/cm^2^) is proportional to the net reaction rate. The experimentally measured *j* is defined by the difference in the current (flux) of the forward *j*_f_ and backward *j*_b_ reactions.

$$j=j_{f}-j_{b}$$ 2

At equilibrium, no net current flows *j* = 0, and currents *j*_f_ and *j*_b_ are equal to the exchange current density *j*_0_. A standard rate of heterogeneous electron transfer, *j*_0,_ is measured at equilibrium. Polarization of the electrode can drive either *j*_f_ or *j*_b_. For a reduction, polarization of the electrode negative of the equilibrium potential *E*_eq_ favors *j*_f_, whereas positive polarization favors oxidation and *j*_b_. Overpotential η defines the electrical polarization relative to *E*_eq_.

$$\eta =E-E_{\mathrm{eq}}$$ 3

*E* is the applied electrode potential. At *E* = *E*_eq_, η = 0, *j* = 0, and *j*_0_ is measured. *j*_0_ is related to the standard heterogeneous rate constant *k* ^0^, which is measured at *E* equal to the standard potential *E* ^0^. Both *j*_0_ and *k* ^0^ are rate constants that characterize the interfacial electron transfer rate.

The relationship between *j* and η is described by the current–potential equation.^12^

$$j(\eta )=j_{0}\left[\frac{C_{O}(\text{0,}\text{η})}{C_{O}^{*}}\exp\left[\frac{-\alpha nF}{RT}\eta \right]-\frac{C_{R}(\text{0,}\text{η})}{C_{R}^{*}}\exp\left[\frac{(1-\alpha )nF}{RT}\eta \right]\right]$$ 4

*C*_O_(0,η) and *C*_R_(0,η) are the concentrations of the oxidized and reduced species immediately at the electrode surface (*x* = 0) at a given η. The concentrations of *O* ^*z*^ and *R* ^*z*–*n*^ in the bulk are *C*_O_^*^ and *C*_R_^*^. All concentrations are in mol cm^–3^. Typically, *n* = 1. Charge transfer coefficient α is a dimensionless parameter that characterizes the symmetry of the energy barrier in the transition state for electron transfer; α partitions the electrical activation energy between the reduction and oxidation. α is constrained to 0 ≤ α ≤ 1. Tafel slopes are inversely dependent on *n*α. *F* is the Faraday constant; *R* is the molar gas constant; and *T* is the system temperature (K). As *f* = *F*/*RT*, at 298.16 K, *f* = 38.92 V^–1^.

At equilibrium where η = 0 or under heavy convection where *C*_O_(0, η) = *C*_O_^*^ and *C*_R_(0, η) = *C*_R_^*^, eq 4 simplifies to eq 5.

$$j=j_{0}[\exp[-\alpha nf\eta ]-\exp[(1-\alpha )nf\eta ]]$$ 5

Eq 5 applies to reaction 1 under constraints of only faradaic current, no mass transport effects, no chemical reactions, all chemical species and electrode materials stable, and electrode surface area known. It is noted that a single transfer coefficient α characterizes the cathodic and anodic reactions, as defined in eq 5.

Eq 5 is the most common form of the BV equation and is the basis for all Tafel analyses. Experimentally, slow polarization of the electrode yields a Tafel plot of log *j* versus η, as illustrated in Figure 1. At sufficiently extreme η, one of the BV terms dominates, and the other becomes negligible. For a cathodic segment where η is sufficiently negative that *j*_f_ ≫ *j*_b_, eq 5 simplifies to the linearized Tafel form for the cathodic branch as shown in eq 6. The constraints to establish the linearization restrict applicability of CTA; the nonlinear regression of Taffit is not subject to linearization constraints of CTA.

$$\log\;j=-\frac{\alpha nf}{2.303}\eta +\log\;j_{0}$$ 6

Historically, this arithmetic simplification enabled the linear graphical analysis of rate data by CTA. Linear regression at sufficiently negative η yields a slope −α*nf*/2.303 that determines α and an intercept of log *j*_0_. At 298.16 K, the cathodic slope is −α*n*[59.16 mV]^−1^. The steeper the magnitude of the slope, the faster the current density changes with η and the higher the α*n*. The intercept at η = 0 is log *j*_0_ that yields *j*_0_, and if *E* ^0^, *E*_eq_, and C* are known, *k* ^0^ is found.^27^ Under BV conditions of eq 4, both branches of a Tafel plot yield the same *j*_0_ and α.

**Figure 1:**
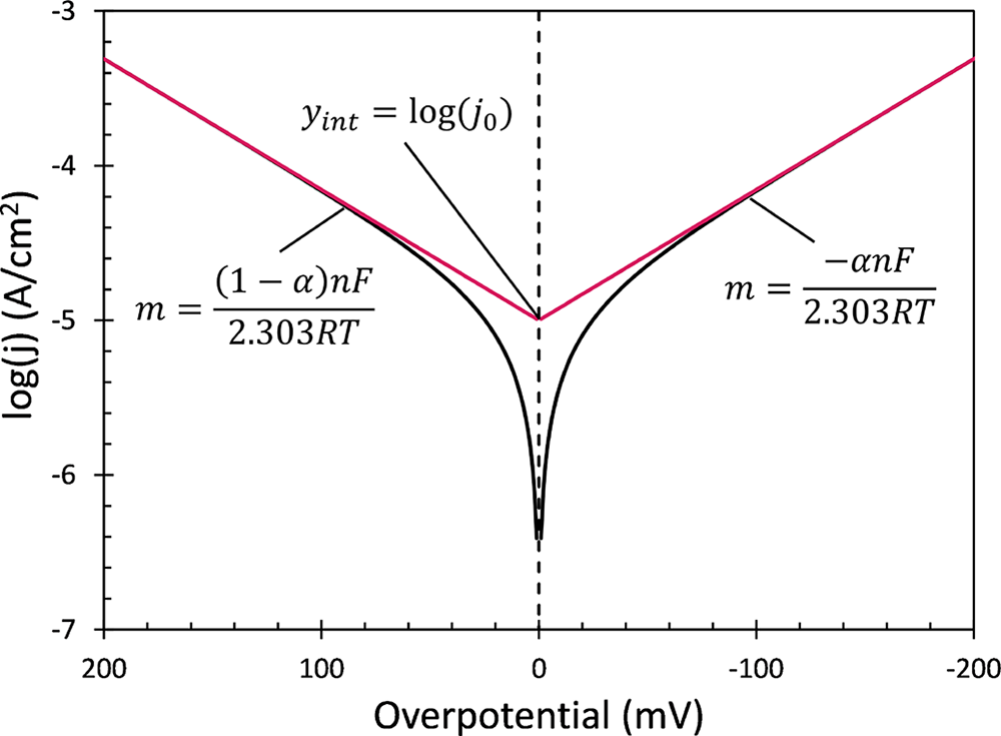
Theoretical representation of Tafel data (black) according to the BV equation and application of CTA linear regression (red) to isolate kinetic parameters. Relevant equations are displayed for the slopes and intercepts of both branches where *j*_0_ is 10^–5^ A cm^–2^ and α is 0.5. Transfer coefficient α, as shown, is the same for both branches. *n* = 1. Within the BV model, log *j*_0_ is the common intercept of both branches at η = 0. Tafel slopes are reported as reciprocal slopes. The Tafel slope for the cathodic branch where η < 0 is −2.303­[*nf*α]^−1^ (mV/decade).

Tafel slopes are often used to report the sensitivity of log *j* to decade changes in η. For η in mV, the cathodic Tafel slope is −2.303­(α*nf*)^−1^ (mV/decade), the reciprocal of the slope in eq 6. At 298.16 K, this value is 59.16­(α*n*)^−1^ (mV/decade). Tafel slopes report the energy (in mV) required to increase the reaction rate by an order of magnitude. Lower Tafel slopes report faster interfacial kinetics.

However, BV kinetics are subject to fundamental constraints on mass transport and η, and CTA is further subject to arithmetic constraints of linearization. Eq 4 is fundamentally specified with no mass transport components to *j*. This sets an upper limit on |η|. Eq 5 is fundamentally specified where |η| is sufficiently small that surface concentrations are unperturbed from bulk concentrations. These constraints are inherent to all Tafel analyses. But, eq 6 for CTA is further arithmetically constrained so that |η| is large enough that *j* is limited by only *j*_f_ (reduction) or *j*_b_ (oxidation). For slow electron transfer rate *j*_0_, these conditions are readily met. Where *j*_0_ is fast, satisfying the constraints on |η| to allow effective CTA is less certain. For fast *j*_0_, the constraints for CTA may not be met or the number of CTA valid data points may be very few. The quality of the data fit by CTA may be compromised, as choice of regression range will substantially impact values found for α and *j*_0_. For fast *j*_0_, CTA may not be valid.

As there is no universally held protocol for choice of a linear regression region, CTA is subject to uncertainty and user bias. Users may choose one arbitrary η region for all analyses that may not satisfy the constraints on |η|. Even within an acceptable |η| region, substantial variation in α and *j*_0_ may result based on choice of CTA linear range. If two different regressions are deemed valid, a researcher is left with a choice as to optimal determination of rate parameters. This introduces variations between researchers. Because parameters detemined by CTA varies with researcher's choice of a linear region, quality and confidence in kinetic parameters determined by CTA is diminished. Comparisons of electrocatalysts are compromised.

Taffit has advantages over CTA. Constraints on mass transport and surface concentrations remain, as in all Tafel analyses. Mass transport sets the upper limit on |η|. However, Taffit fits both branches of the Tafel plot, according to eq 5. Taffit relaxes constraints as η → 0 to yield *j*_0_ and a single α. The Taffit algorithm is implemented in Excel to improve precision and accuracy of determined kinetic parameters. Taffit substantially suppresses user bias.

## Taffit Design

An operational schematic for the design of Taffit is shown in Figure 2. Taffit imports, processes, and fits experimental data to eq 5. This yields *j*_0_, α, and Tafel slopes while eliminating a user-selected linear range. Taffit imports a single LSV data file (current *i* vs *E*), or alternatively, an existing Tafel plot file (log *j* versus η). Either .txt and .csv files with different data structures, delimiters, and header information can be imported. Taffit converts data into Tafel form and isolates values within η_win_, a user defined window around η = 0. For example, η_win_ = 60 mV fits data ± 30 mV of η = 0. η_win_ and *n* are the only user-defined parameters in Taffit. Most typically, *n* = 1 and 20 ≲ η_win_ ≲ 180 mV. Implementation of the Taffit tool is detailed in the Supporting Information. The Excel macro file Taffit.xlsm and an example text data file are also in SI.

**Figure 2:**
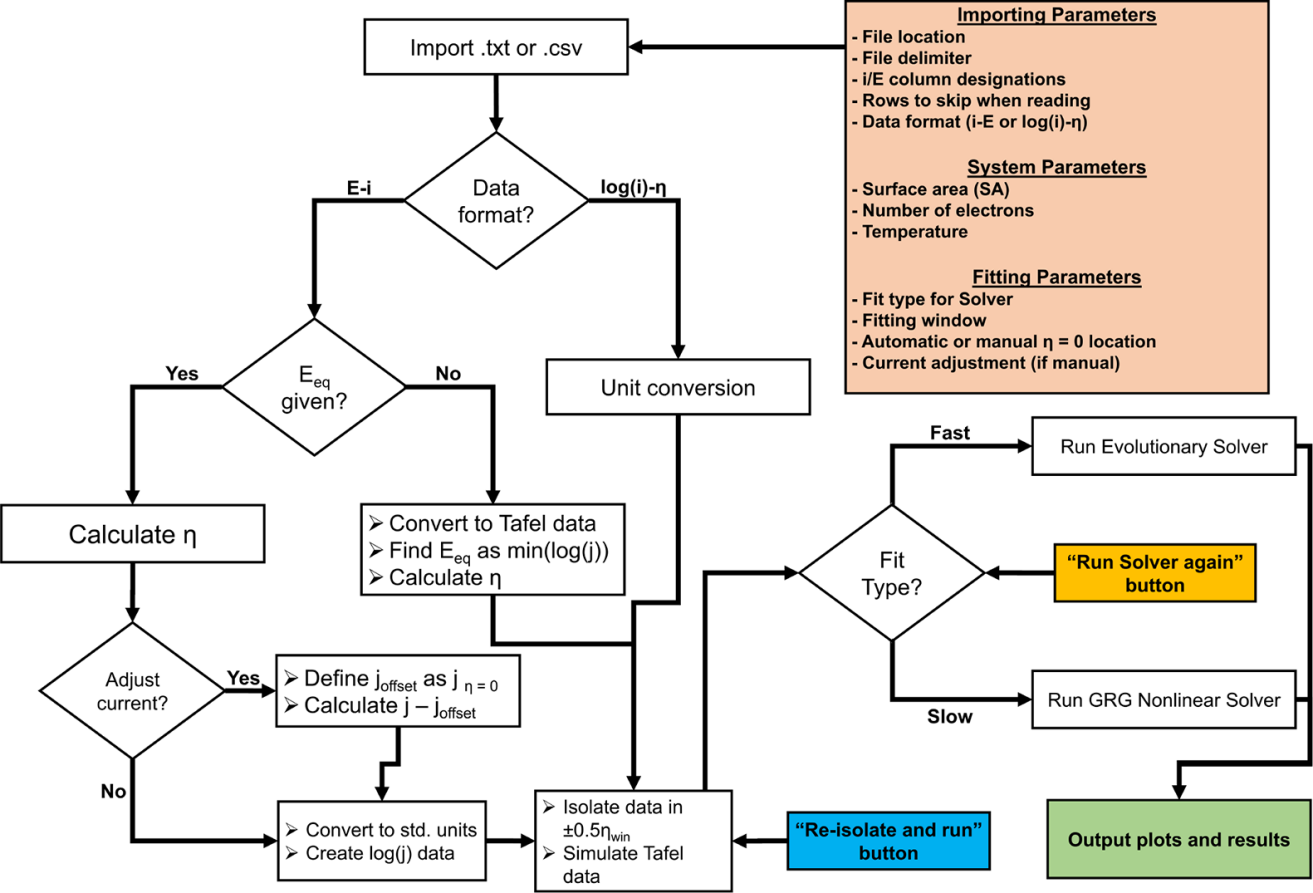
Flowchart of Taffit design and operation. Uncolored boxes represent operational steps, and diamond boxes represent conditional statements. The orange box represents initial inputs to the function, and the green box represents the function completion. Blue and gold boxes represent the entry points for the function.

Tafel data isolated by η_win_ undergo nonlinear regression based on eq 5. Fit quality is assessed as the standard error of the estimate σ.

$$\sigma =\sqrt{\frac{\sum (\log\;j_{\exp}-\log\;j_{\mathrm{BV}}{)}^{2}}{N-2}}$$ 7

*j*_exp_ is the experimentally measured current and *j*_BV_ is the current fit to eq 5 at the same overpotential. *N* is the total number of data points being fit.

The Solver tool in Excel is called to find the conditions of the lowest σ and reports the *j*_0_, α, and equivalent Tafel slopes of closest fit. Best fits minimize σ, where σ ≲ 0.1 characterizes a sufficient fit within Taffit. Slightly higher values of σ may be adequate, although the fit is of lower statistical quality. For σ ≫ 0.1, the plot of the fit overlaid on the experimental will be obviously incorrect. Users are provided results, graphical data, and macro buttons to rerun the analysis (Figure 3).

**Figure 3:**
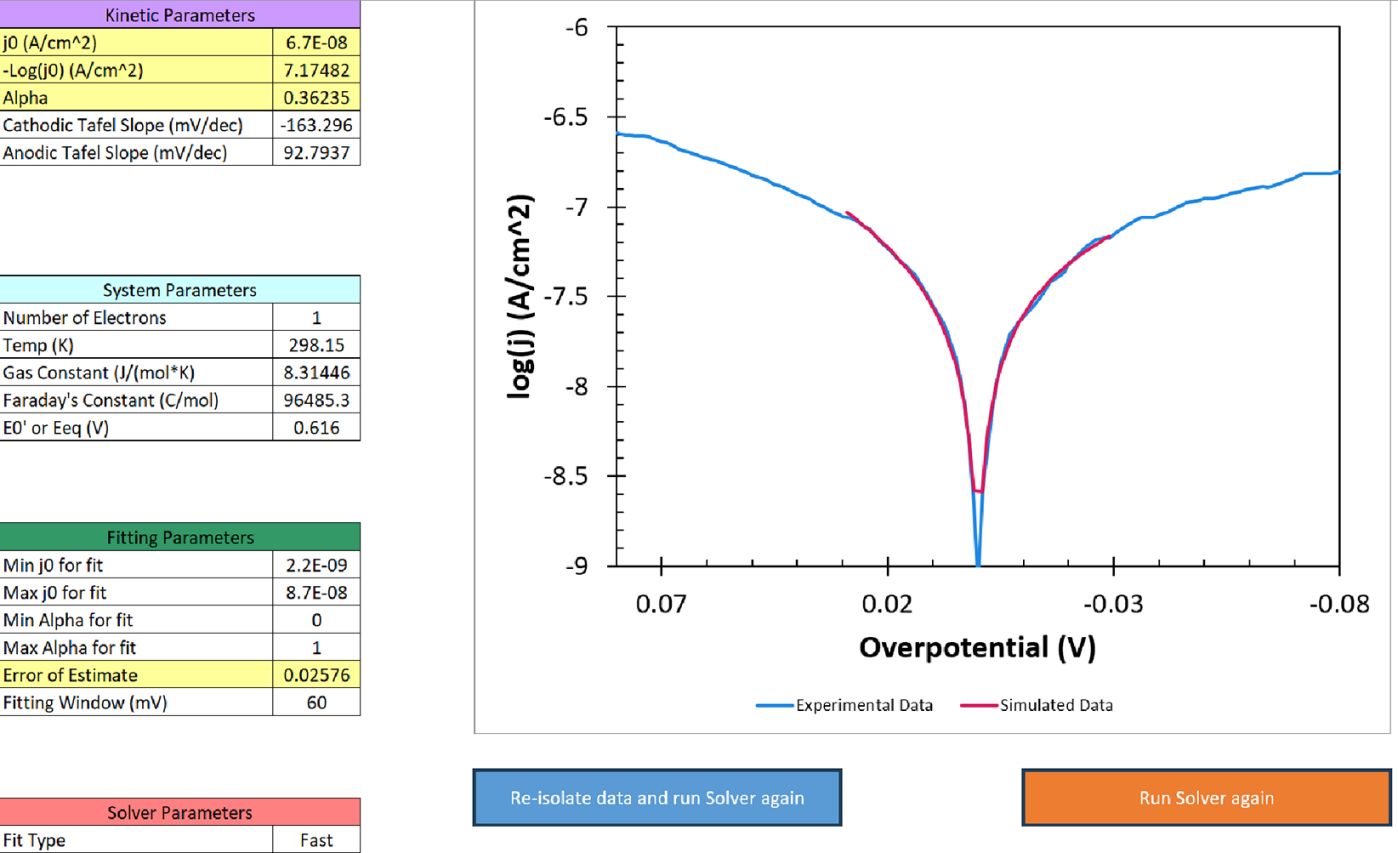
Example of the Taffit fitting results and kinetic parameters. Shown are HER Tafel data for 0.45 cm^2^ glassy carbon at 5 mV s^–1^ in H_2_ sparged and stirred 0.5 M H_2_SO_4_. The blue button isolates data again and is used for changing the fitting window η_fit_. The gold button simply runs Solver again on the η_win_ isolated data.

Two different Solver optimization algorithms can be called in Taffit. The Evolutionary solving method is best suited for Tafel data and can obtain results in as little as ten seconds. This is the fast Fit Type. The GRG Nonlinear method is the slow Fit Type but can be used to more confidently locate the values for the greatest optimization. Installation and operational guides, VBA (Visual Basic) code, and additional instructions are available in the Supporting Information.

Taffit is designed to mitigate subjectivity in CTA. The tool removes linear regression over an ill-defined range selected by the user. Constraints on small η ranges are removed to include more data as η → 0. As in all Tafel analyses, Taffit does not account for mass transport observed at higher currents and so remains limited at high |η| ranges, where mass transport limits faradaic current. Taffit applies to faradaic current of the BV equation. *iR* corrected data are especially important for high *j*_0._ The Taffit tool fits data over a wider overpotential range and utilizes more data from the Tafel plot than CTA.

## Advantages, Limitations, and Caveats of Taffit

There are limitations inherent to all Tafel analyses, as Tafel analysis is based on the BV equation. The BV equation, eq 5 is derived under specific constraints common to all Tafel analyses. CTA and Taffit are Tafel analyses. The linearization of CTA imposes further constraints as η → 0. Taffit relaxes the CTA constraints by nonlinear regressions about η → 0. Here, the limitations of Tafel analyses, Taffit advantages, and caveats and notes for Taffit application are outlined.

### Tafel Analysis Limitations

The constraints embedded in Tafel analysis arise through the assumption used to derive the BV equation. Tafel analysis applies to faradaic current only. Correction for uncompensated solution resistance is especially important where electron transfer is fast, log *j*_0_ ≳ −4. In the simplification of the current potential equation (eq 4) to the BV equation (eq 5), concentrations at the electrode surface are set to bulk concentrations. This requires that there is no mass transport limitation to the current, which sets an upper limit on |η|. At sufficiently extreme overpotentials, electron transfer rates increase to become competitive with mass transport rates; under these conditions, faradaic current is limited by both mass transport and electron transfer. Tafel analysis is not applicable at these extremes of overpotentials.

In the form of eq 5, Tafel analysis requires no chemical reactions. The concentrations of O^*z*^ and R^*z*–*n*^ are unchanged at overpotentials valid for Tafel analysis. The electrode and electrocatalyst are chemically and electrochemically stable. Eq 5 does not account for surface coverage or adsorption of the redox species or oxide layers on the electrode and electrocatalyst. Tafel analysis does not include multistep reactions beyond simultaneous electron transfers where *n* > 1.

Eq 5 is characterized by a single value of log *j*_0_ and one value for α. These values are common to both the cathodic and anodic branches of the Tafel plot.

Tafel analysis characterizes the electron transfer and does not differentiate between an electrode and an electrocatalysts. However, the surface area of the electrode and electrocatalyst are needed to evaluate the current density and so *j*_0_. For a flat electrode or a monolayer of finely divided catalyst on the electrode, the electrochemical surface area (ECSA) is easily evaluated. Where the electrocatalyst is a particulate, the correct ECSA is critical to determination of *j*_0_. If ECSA is underestimated, *j*_0_ is overestimated. In comparison of different electrocatalysts, well determined *j*_0_ is essential.

CTA and Taffit are both Tafel analyses constrained by the assumptions used to derive the BV equation. Tafel analysis excludes mass transport limitations to the faradaic current, which sets an upper limit on |η|. CTA is further constrained as η → 0 because the linearization restricts the analysis to only the cathodic or anodic branch of the Tafel plot. In CTA, a lower limit, |η| is set algebraically. Linear ranges selected by the user in CTA are subject to user bias. Where *j*_0_ is fast, a valid range of overpotential may not be accessible or the number of valid data points may be too few to allow competent evaluation of *j*_0_.

### Advantages of Taffit over CTA

Taffit, like all Tafel analyses (eq 5), does not include mass transport limitations, which sets an upper limit on |η|. Taffit has advantages over CTA as Taffit exploits data about η = 0. Whereas CTA is limited by constraints for linear regression, Taffit uses nonlinear regression to fit both branches of the Tafel plot, including data about zero overpotential. Taffit statistically vets the quality of the fit through the standard error σ. Minimized σ identifies the best fit of the data.

Advantages of Taffit over CTA are several. User bias of CTA associated with selecting a linear region is eliminated. Taffit is not subject to a lower limit on |η|, so more data points are available to fit of the data with improved statistics. Taffit allows assessment of fast *j*_0_ where CTA may fail.

### Caveats and Notes for Taffit Application

Taffit is subject to all the limitations for Tafel analyses, as outlined above. Two main constraints are that |η| is limited to prevent mass transport effects and that the redox species, the electrode, and any electrocatalyst are chemically stable.

Taffit provides best fit of log *j* versus η to yield a single log *j*_0_ from the intercept, and a common α from the slopes. Tafel slopes derive α. Taffit does not remove the user’s responsibility to critically evaluate the parameters output by Taffit. For example, if the electrocatalyst or electrode undergoes chemical change or forms an oxide layer, Taffit will provide a statistically appropriate fit to the data, but interpretation of the mechanism and chemistry will fail.

#### Taffit, Experiments, and Mechanisms

Taffit does not address multistep mechanisms that include adsorption and chemical steps. Taffit does allow serial electron transfer where *n* > 1, consistent with eq 5. If Taffit returns α > 1 when run with *n* = 1, then try *n* = 2. This is the only adaptation for more complex reactions. For more complex mechanisms, such as oxygen reduction (ORR) and oxygen evolution (OER) reactions, Taffit output should be evaluated cautiously. Where the first or second electron transfer is rate determining and other constraints including no chemical steps are met, Taffit may apply to complex reaction sequences. If conditions are not met for Taffit, Taffit output parameters are statistically relevant, but physical interpretation of the results is subject to further analysis in light of the mechanism(s).

An appropriate measurement protocol is to measure the open circuit potential (OCP), the equilibrium potential against which η is measured (eq 3). For a reduction such as the hydrogen evolution reaction (HER), undertake a slow scan rate LSV experiment scanning potential from +20 to −100 mV relative to OCP. Collect current relative to η. Best practice is to have both the oxidized and reduced forms in the electrolyte to establish *E*_eq_. For HER, this would be H^+^ and saturated H_2_. In the same solution, either measure the uncompensated resistance by a small (50 mV) step in the nonfaradaic region or use resistance compensation available on the potentiostat.

Resistance compensation is especially important where log *j*_0_ ≲ −4. At high *j*_0_, a few ohms of uncompensated resistance will underestimate *j*_0_. For low *j*_0_, resistance compensation is less critical. For measurements at small electrodes, total current is lower and correction for solution resistance are diminished. Details for correcting the total measured current to the faradaic current using measured solution resistance are available in Chapter 6 of *Electrochemical Methods*.^12^ Where nonfaradaic resistance and capacitance are measured, the charging current measured as capacitance evaluates ECSA well, Chapter 1 of *Electrochemical Methods*.^12^ ECSA is critical in evaluating particulate electrocatalyst.

#### Taffit Parameters

Some comments about Taffit parameters are noted.

##### Electrochemical Surface Area (ECSA)

Good measurement of ECSA is critical to correct determination of *j*_0_. Current density *j* is found by normalizing by ECSA. For particulate electrocatalysts, the ECSA can be substantially greater than the geometric area of the electrode. Where ECSA is underestimated, *j*_0_ and efficacy of the electrocatalyst are overestimated. Taffit does not determine ECSA.

##### Scan Rate, *v*

Mass transport rate increases with scan rate. For fast electron transfer rates, an increase in *v* may better resolve *j*_0_. However, as v increases, current increases, and adequate resistance compensation is critical.

##### Limits on α

Tafel slopes and the transfer coefficient α are inversely related; Tafel slope for the cathodic branch is −2.303­[*nf*α]^−1^ (mV/decade). Eq 5 constrains *n*α to between 0 and 1. If Taffit run with *n* = 1 returns α > 1, rerun Taffit with *n* = 2. If Taffit yields α of 1 or 0, or log *j*_0_ equal to the maximum experimental log *j*, or σ ≫ 0.1, the fit is likely not valid and Taffit should be rerun with different constraints and a Fit Type of slow to determine if a better fit is found. Defined in eq 5, α is constrained as 0 ≲ α ≲ 1. If on visual inspection, the Tafel plot is fairly symmetric, an α of about 0.5 ± 0.2 is expected; if the plot is asymmetric, more extreme α is expected. Where the Tafel plot is symmetric and log *j*_0_ ≲−4, the analysis is typically straightforward; otherwise, attention to the mechanistic interpretation of the fit is needed.

##### Standard Error of the Estimate, σ

σ is a statistical characterization of the quality of the fit to the data. In general, σ ≲ 0.1 is taken as a good fit. Similar values of σ across several η_win_ are anticipated for well fit data. Slightly higher σ values may mark an adequate fit to the data, but the fit warrants review. Visual inspection of the fit on the experimental Tafel plot may be useful. In cases of valid but slightly higher σ, CTA is unlikely to yield well determined kinetic parameters. As the number of samples *N* increases (eq 7), σ tends to decrease. *N* tends to increase with wider η_win_, but η_win_ is capped to avoid mass transport effects.

##### Overpotential Window, η_win_

If η_win_ is well selected, then variation in log *j*_0_ and α with η_win_ should be minimal. Restrict η to avoid mass transport effects captured in wider η_win_. Mass transport impacts are more readily observed with wider η_win_. Common choices for η_win_ are between 30 and 180 mV.

## Experimental Methods

Taffit is vetted against experimental data for HER (2H^+^ + 2*e* ⇌H_2_) on glassy carbon (GC), platinum, and nickel electrodes and literature data for several metal phosphide and selenide electrocatalysts.

Outcomes for Taffit and CTA are compared. Taffit requires the user to input η_win_. η_win_ selects the potential window over which Taffit optimizes fit to eq 5, where η_win_ includes η = 0. Unlike CTA, Taffit has no lower boundary for |η|. The upper limit on η_win_ is set by the onset of mass transport impacts on j, consistent with exclusion of mass transport in all Tafel analyses (eq 5). To vet Taffit, a range of η_win_ values were evaluated. η_CTA_ identifies the potential window over which the data are fit by CTA. Taffit is also benchmarked against literature data that were rigorously collected with CTA.

### Precision Comparisons for Experimental HER Data

Tafel plots for the hydrogen evolution reaction (HER, 2H^+^ + 2*e* ⇌ H_2_) were collected experimentally for precision comparisons. Experiments were conducted in a H_2_-sparged and stirred 0.5 M H_2_SO_4_ solution. Electrode potential was swept slowly in linear sweep voltammetry, LSV, on a BASi potentiostat from positive of the open circuit potential (OCP) through OCP and to negative of OCP. OCP, measured at equilibrium before the LSV, is the equilibrium potential *E*_eq_. Data are not corrected for nonfaradaic resistance (*iR*) drop. The three-electrode setup consisted of a 0.45 cm^2^ Pine Instruments GC or platinum working electrode, a CHI saturated calomel reference electrode, and a graphite rod counter electrode. For the single measurement of HER on nickel, a 0.07 cm^2^ BASi nickel electrode is used. Working electrodes were polished in alumina slurries of successively finer grit (1, 0.3, and 0.05 μm) and thoroughly rinsed prior to analysis.

Effects of η_win_ and η_CTA_ analysis windows were compared for the same set of Tafel plots. Regression was performed for the cathodic branch in a rolling η_CTA_ window, and Taffit analyses were performed using increasing η_win_ values. The resulting kinetic parameters and their relative standard deviations were compared.

### Literature Data for HER

Literature Tafel data were also used to demonstrate Taffit. Representative Tafel plots or LSVs were used for data analysis, where available. Materials science research typically produces Tafel plot segments; these are not full Tafel plots. Tafel segment plots contain only linear segments of the Tafel plots. Available regions were fit using Taffit and the resulting kinetic parameters; commonly, Tafel slopes were compared.

## Results and Discussion

Taffit is first applied to experimental data collected for this study. The purpose is to demonstrate that Taffit increases precision and decreases user bias. Measurements are made for HER at electrodes with fast and slow kinetics. Taffit is also used to fit Tafel data available in the literature. Tafel slope comparisons are used for validation for Taffit. A first report of kinetic parameters for HER on glassy carbon is made.

### Precision Comparisons

Two electrode materials, Pt and GC, were chosen for comparing algorithmic Taffit and classical CTA analyses. Pt and GC electrodes present kinetic extremes for HER catalysis. See Figure 4. The overpotential needed to drive HER on Pt is minimal. Reductive overpotentials of no more than a few tens of millivolts result in HER current onset. Conversely, overpotentials in excess of −0.9 V are required to drive HER on GC. Precision for Taffit and CTA is compared for the two extreme cases.

**Figure 4:**
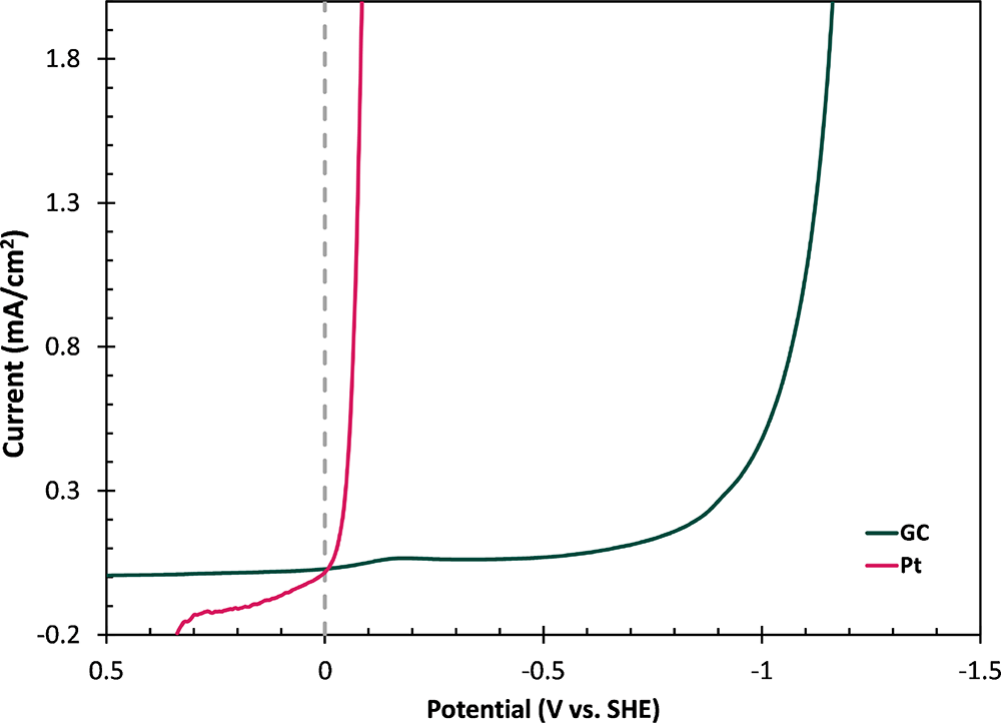
Representative LSV curves for HER on Pt (red) and GC (green) disk electrodes in H_2_ sparged and stirred 0.5 M H_2_SO_4_, collected at 100 mV/s. The thermodynamic HER redox potential (dashed gray line) is included for reference.

To avoid user bias, direct comparison of experimental kinetic parameters from Taffit and CTA is not appropriate. As choice of regression region can significantly impact kinetic parameters determined by CTA, user bias toward agreement between Taffit and CTA is introduced. To avoid bias, comparisons of the precision are more appropriate. Averages and standard deviations are reported.

#### HER on Pt

Figure 5 shows Taffit and CTA results for a single representative Pt scan. HER kinetics on Pt are fast, and deviations from BV are expected to onset at low |η| as mass transport and electron transfer rates both limit *j*. In Figure 5 top, Taffit yields a narrow range of log *j*_0_ with η_win_ values of 60, 120, and 240 mV, with slightly higher log *j*_0_ with wider η_win_, as expected as mass transport contributions to *j*. Impacts of η_win_ on measured log *j*_0_ are not substantial, as show in Figure 5. In Figure 5 bottom, CTA for HER yields more substantial deviations in slope as the user selected linear regression range η_*CTA*_ changes. No valid overpotential window η_CTA_ is reached at Pt because well before |η| required for *j*_f_ ≫ *j*_b_ is reached, sufficient H_2_ is generated to violate constraints on *C*(0, η) equal to C*. Choice of η_CTA_ has a profound effect on evaluation of log *j*_0_ from the intercept and α from the slope. No CTA regression produces a common *j*_0_ with the anodic branch. This illustrates impacts of user bias in CTA.

**Figure 5:**
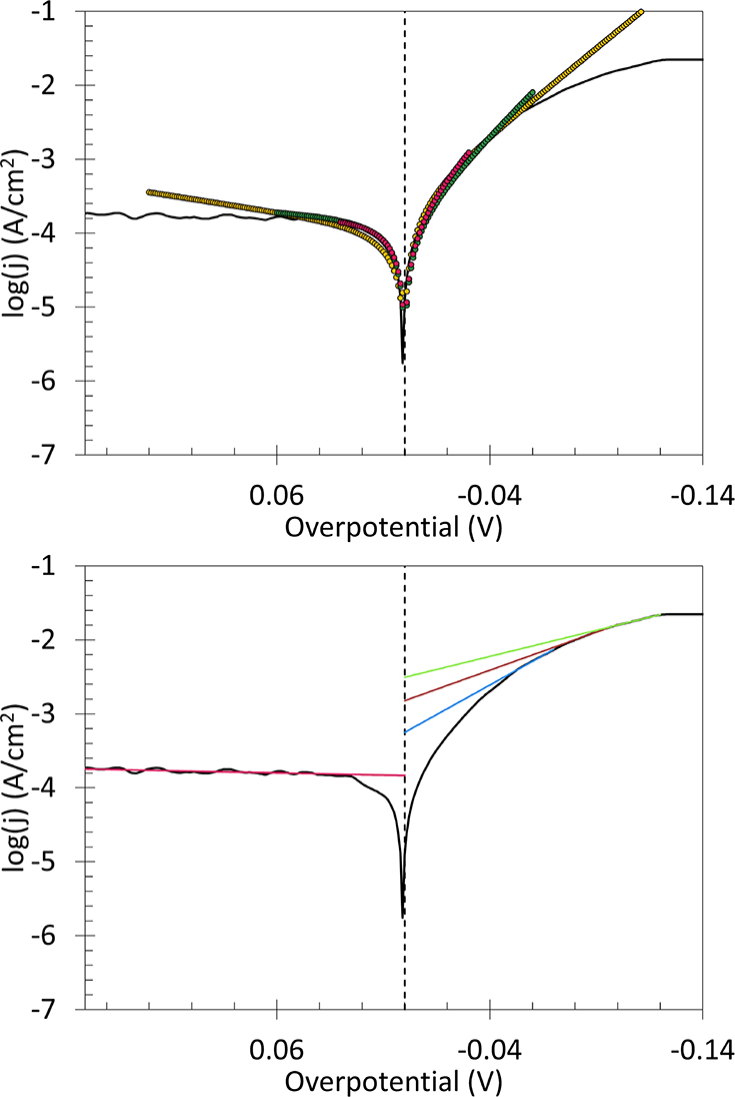
Representative Tafel data (black lines) for HER on 0.45 cm^2^ Pt in H_2_ sparged and stirred 0.5 M H_2_SO_4_ recorded without *iR* compensation at 5 mV s^–2^. Top: Algorithmic, Taffits (colored circles) across different fitting windows, η_win_. Shown are fits in 60 (red), 120 (green), and 240 mV (yellow) windows around η = 0 (dashed line). Bottom: Select linear regressions (colored lines) for the same Pt Tafel plot. Shown are linear regressions for the anodic segment (red) as well as cathodic regressions (blue, green, and yellow) for different η_CTA_. CTA slopes identify different values of α and the intercepts identify different values of log *j*_0_.

The advantages of Taffit are most apparent in cases of fast kinetics, as on Pt. Data analysis for HER on Pt is shown in Table 1 for Taffit and CTA. Although still restricted by BV mass transport constraints, variation from subjectivity in η is markedly lower for Taffit. From the Table, the relative standard deviation RSD for *j*_0_ is 65% for CTA and 13% for Taffit. The precision of log *j*_0_ is markedly higher for Taffit.

**Table 1:** Taffit and CTA Precision Comparisons for HER Tafel Plots^a^

electrode	–log (*j*_0_)	*j*_0_ (A/cm^2^)	α	Tafel slope (mV/dec)
Pt (CTA)^b^	2.9 ± 0.3	(2 ± 1) × 10^–3^	0.3 ± 0.1	–100 ± 30
	10%	65%	33%	33%
Pt (Taffit)^b^	3.89 ± 0.06	(1.3 ± 0.2) × 10^–4^	0.83 ± 0.08	–36 ± 3
	1.5%	13%	9%	9%
GC (CTA)	7.00 ± 0.07	(1.0 ± 0.2) × 10^–7^	0.16 ± 0.03	–400 ± 70
	1.1%	17%	19%	19%
GC (Taffit)	7.16 ± 0.02	(6.9 ± 0.3) × 10^–8^	0.35 ± 0.03	–170 ± 20
	0.30%	5%	11%	11%

a Data are not *iR* corrected.

b *n* = 2 was used for calculations

The commonly reported literature log *j*_0_ value for HER on Pt is −3.^28^ The experimental data reported here are not corrected for the uncompensated resistance. For high electron transfer rates, a few ohms of uncompensated solution resistance readily deppresses the experimentally determined *j*_0_.^29,30^ Where *j*_0_ is low, resistance to charge transfer is larger and the uncompensated resistance has relatively less or negligible impact on measured *j*_0_.

An important parameter when using Taffit to evaluate α is *n*, the number of electrons. It is typically assumed that *n* = 1 for electron transfer reactions. However, *n* = 2 is common for HER on Pt in water. *n* = 2 is not uncommon for fast reactions where one electron transfer is fast, and the other is slow. For HER on Pt, the apparent *n* is a simultaneous two-electron transfer. With *n* = 1, Taffit yields a transfer coefficient of 1.97, which is outside the range between 0 and 1 expected for partition of the free energy of activation in the transition state. Taffit is run again to evaluate *n* = 2. For *n* = 2, the precision in α and the corresponding Tafel slope is 33% for CTA and 9% with Taffit. In the table, α values of 0.3 and 0.83 are reported for CTA and Taffit. In Taffit, the common α value leads to (1 – α) = 0.17 for the anodic branch, consistent with greater sensitivity to η for HER than the hydrogen oxidation reaction (HOR).

On Pt for *n* = 2, the expected HER Tafel slope is 30 mV/dec, consistent with fast electron transfer. For HER at Pt microelectrodes and detailed data analysis, a Tafel slope of 30 mV/decade is reported in 1 M HClO_4_.^11^ Here, Taffit yields −(36 ± 3) mV/decade. As no valid region exists for CTA, the Tafel slope of −(100 ± 30) mV/decade is not applicable. Tafel slope of 30 mV/dec identifies Volmer Tafel kinetics on Pt.^31^ In Volmer Tafel kinetics, two H^+^ ions are reduced to H^•^ adsorbed on Pt to rapidly yield H_2_. Taffit exploits data in the low |η| range to efficiently evaluate kinetic parameters of *j*_0_, α, and Tafel slope with good precision. Application of Taffit to HER on Pt statistically validates the fit of log *j* versus η, but the physical significance of the determined parameters relies on assessing the HER mechanism specific to Pt.

#### HER on Glassy Carbon

GC is a poor electrocatalyst for HER. Slow kinetics for HER on GC are apparent in Tafel plots with lower log *j*. CTA is more readily applied with slower interfacial kinetics than for fast kinetics. CTA and Taffit results are presented in Figure 6. In CTA where multiple η_*CTA*_ ranges are valid, variation in log *j*_0_ and α is still observed based on the user choice of the linear region. No common log *j*_0_ and α are identified for the two branches by CTA. Only minor variation with η_win_ is noted for Taffit as η_win_ increases. Taffit yields common log *j*_0_ and α.

**Figure 6:**
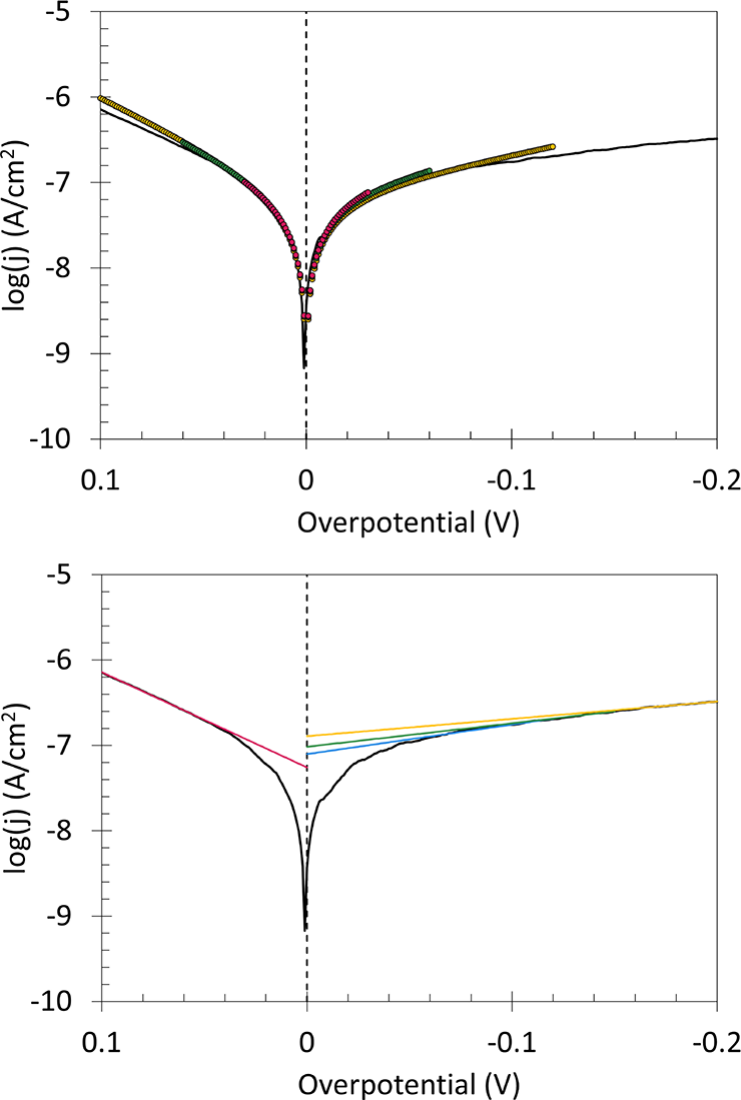
Representative Tafel plot for HER on 0.45 cm^2^ GC in H_2_ sparged and stirred 0.5 M H_2_SO_4_, measured without *iR* compensation at 5 mV s^–1^. Top: Algorithmic Taffit (colored circles) across different fitting windows. Shown are fits in 60 (pink), 120 (green), and 240 mV (yellow) windows around η = 0 (dashed line). Bottom: Select linear regressions (colored lines) for the same Tafel plot. Shown are linear regressions for the anodic segment (pink) as well as cathodic regressions (blue, green, and yellow). Common log *j*_0_ and α are not found by CTA.

Data analysis for HER on GC is summarized in Table 1, where *n* = 1. Precision is higher on GC than on Pt because log *j*_0_ is lower on GC. For lower *j*_0_, relative impacts of uncompensated resistance on reported parameters are lesser. Precision for *j*_0_, α, log *j*_0_, and Tafel slope is higher for Taffit. The data are better fit by Taffit than CTA. Comparable log *j*_0_ and *j*_0_ values are found for the two methods. However, α values and Tafel slopes differ substantially. The Tafel slope determined by Taffit is closer to expected HER benchmarks with lower RSD. Mechanisms are nominally classified as Tafel, Heyrovsky, and Volmer steps based on Tafel slopes of 30, 40, and 120 mV/dec.^31^ An HER Tafel slope of −170 mV/dec is nominally consistent with Volmer kinetics, perhaps impacted by the surface coverage. In Volmer kinetics, the rate-determining step is a single electron transfer from an electrode atom to protons to form adsorbed H^•^.

#### HER on Nickel

Rates of HER on nickel are intermediate between Pt and GC. A screenshot of Taffit for H_2_ sparged 0.5 M H_2_SO_4_ at a 0.07 cm^2^ nickel electrode is shown in Figure 7. The equilibrium potential *E*_eq_ was measured. The fitting window, η_win_ = 90 mV. Values were invariant for η_win_ between 20 and 120 mV for the single replicate electrode. Taffit yields log *j*_0_ = −4.6; the error of estimate, σ = 0.017, which is adequate. Taffit yields *j*_0_ about 5-fold higher than reported by Trasatti.^28^ Given the single replicate and limited precision of the electrode area, log *j*_0_ values are not dissimilar. From Taffit, the transfer coefficient α = 0.24 for *n* = 1, consistent with asymmetric partition of energy in the transition state for HER. This is reflected in the distinct cathodic and anodic Tafel slopes of −243 and 78 mV/dec. Identification of the linear range for CTA is difficult because log *j*_0_ is moderately high, and Tafel slopes on approach to minimum η are steep.

**Figure 7:**
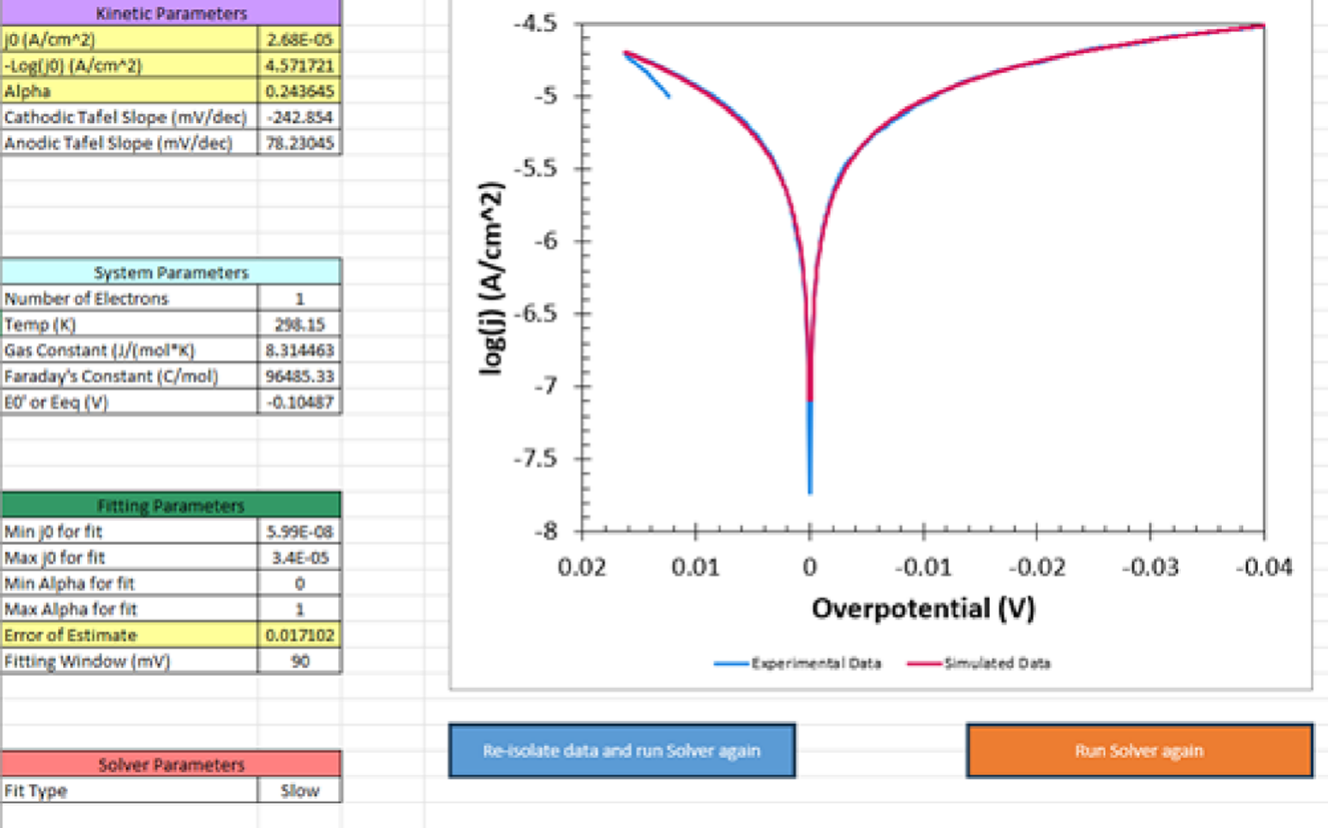
Screenshot of Taffit for HER on 0.07 cm^2^ Ni electrode in H_2_ sparged and stirred 0.5 M H_2_SO_4_, measured without *iR* compensation at 1 mV s^–1^. Potential range for LSV is +20 mV to −100 mV relative to the open circuit potential (OCP). Taffit (red) overlays the experimental data presented as a Tafel plot. The fit type was slow over η_win_ = 90 mV. log *j*_0_ = −4.6. Similar results were found for other fit types and η_win_ ranges.

In summary, data and precision analyses are shown in Table 1 for both fast (Pt) and slow (GC) electron transfer kinetics. Taffit offers decreased analysis time and better precision. Precision is higher for Taffit for all parameters under conditions of both fast and slow kinetics; kinetic parameters are as anticipated for HER measurements. For fast kinetics on Pt, no fundamentally and arithmetically valid linear range is available for CTA. Because Taffit exploits data at low |η| and fits both log *j* branches to eq 5, Taffit fits high rate data. Taffit finds the single α that yields the same log *j*_0_ for the anodic and cathodic branches. Although the same exchange current densities are measured by CTA and Taffit for slower electron transfer rates on GC, the range of η_*CTA*_ includes several valid but distinct slopes for log *j* versus η. Taffit yields more precise values of *j*_0_ and both more accurate and precise values of α and the Tafel slope. For Pt, Taffit statistically confirmed the quality of the fit of log *j*_0_ versus η, but review of the *n* = 2 mechanism was required to interpret the results. Taffit for GC and Pt indicate HER mechanisms limited by Volmer and Tafel steps, respectively. For a single replicate for HER on Ni, Taffit finds log *j*_0_ = −4.6, α = 0.24, and cathodic and anodic Tafel slopes of −243 and + 78 mV/dec, within an acceptable σ over a range of η_win_.

### Literature Comparisons

Comparisons were made between Taffit and CTA for HER using different electrocatalyst materials. Metal phosphides, carbon supported platinum, and cobalt derived catalysts reported in the literature were used for comparisons.^32,33^ Full Tafel plots or LSVs were used where possible. If full plots were unavailable, representative Tafel segments were used (Figure 8). Of note are the reversed axes used in plotting the literature data, η versus log *j*. This is common in catalyst studies for easier determination of the Tafel slope in mV/dec, where log *j*_0_ is roughly estimated from the intercept as η → 0.

**Figure 8:**
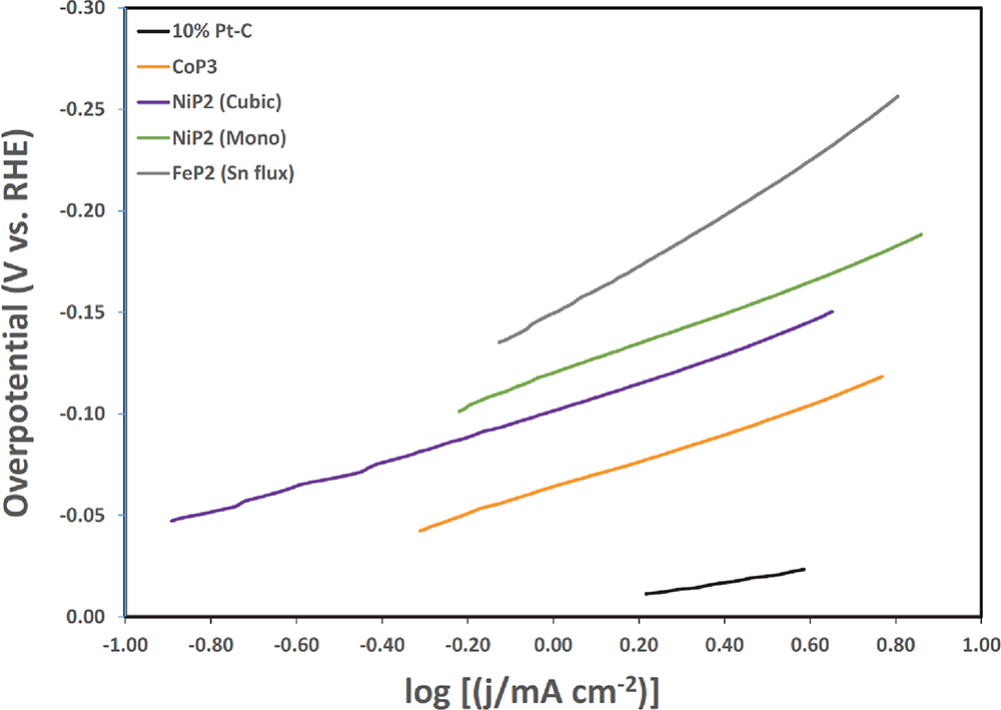
Representative HER Tafel plot segments for several different electrocatalysts in 0.5 M H_2_SO_4_. Reprinted with permission from ref 32. Copyright 2019, American Chemical Society.

Taffit results and comparisons to the literature Tafel data are shown in Table 2. The literature data were analyzed by CTA. Exchange current densities are not commonly reported in materials studies. Comparisons are made to reported Tafel slopes.

**Table 2:** Comparisons between Taffit and CTA for Literature Tafel slopes

catalyst	Taffit slope (mV/dec)	lit. slope (mV/dec)	percent relative difference (%)
CoP_3_	–70.0	–78 ± 2	11
FeP_2_	–128	–129 ± 11	0.85
NiP_2_ cubic	–66.9	–91 ± 4	31
NiP_2_ mono	–77.2	–81 ± 2	4.8
10% Pt-C^a^	–65.5	–34 ± 2	63
Co_0.5_Ni_0.5_Se_2_ ^a^	–57.4	–55	4.3
CoSe_2_ ^a^	–42.8	–47	9.4

a *n* = 2 was used in Taffit analysis.

In the majority of the comparisons made, the literature CTA and the algorithmic Taffit Tafel slopes show good agreement. With the exception of 10% Pt-C, the differences in the Tafel slopes are not sufficient to change the interpretation of the data as Volmer, Tafel, or Heyrovsky limited. Taffit is able to reproduce the Tafel slope from representative segments of Tafel plots. The greatest difference between the two methods is for 10 % Pt-C. It is not surprising that the largest discrepancy would be reported for a system with fast kinetics. As described above, regression of Pt HER Tafel plots is a difficult procedure. Small changes in the CTA regression range can produce substantially different kinetic parameters. The representative Pt-C plot in Figure 8 involved regression across a small range of low η values, which can significantly diminished the quality of CTA data analysis. In general, for these literature data, correlation between CTA and Taffit is good.

## Conclusions

Taffit provides algorithmic analysis of Tafel data to evaluate kinetic parameters for interfacial electron transfer of log *j*_0_, *j*_0_, α, and Tafel slopes. As compared to fits by classical means (CTA), Taffit provides higher precision and mitigates user bias. As in all Tafel analyses, Taffit and CTA follow BV kinetics that preclude mass transport effects. Taffit data are analyzed without the user identified linear regions of CTA. Taffit minimizes standard error in fit to the BV equation (eq 5) in an overpotential window η_win_ about η = 0. Taffit fits both anodic and cathodic branches of the Tafel plot and finds log *j*_0_ and α common to both branches, as specified by the BV equation. Cathodic and anodic Tafel slopes are found.

The Taffit algorithm is deployed in Microsoft Excel. Taffit is designed for ease of use by nonexperts. Analysis is fast and efficient to determine *j*_0_, α, and equivalent Tafel slope by closest fit to the BV equation by minimizing the standard error of the estimate. Taffit offers several advantages over the more conventional CTA. Principally, the tool shows greater precision and substantially decreases user bias inherent to CTA. Taffit utilizes data as |η|→ 0 to facilitate measurements for faster kinetics. Taffit statistically vets the fit of log *j* versus η, but interpretation of the fitting parameters should be reviewed in light of the mechanism. It should be confirmed that the conditions for all Tafel analyses are meet in interpretation of Taffit results.

Taffit is vetted against experimental data for HER on Pt and GC electrodes. Easily deployed, Taffit provides a first report for HER on glassy carbon at pH 0 under H_2_ as log *j*_0_ of −7 and Tafel slope of −170 mV/decade, for *n* = 1. Good agreement is observed between values reported in the literature and those fit by Taffit. Knowledge of specific mechanistic properties and characteristics of the system is not necessary for the use of Taffit. The developed tool is generalized and applicable to various electrochemical systems.

## Supplementary Material



## Notes

An operational guide, VBA code, and representative LSV data for HER on glassy carbon (PDF)
