# Supplementary material for: Taffit: An Excel Tool for Fitting Tafel Data
Source: ACS Meas Sci Au. 2025 Jul 15;5(4):536–46. doi: 10.1021/acsmeasuresciau.5c00038 (PMC12371579; doi:10.1021/acsmeasuresciau.5c00038)
Supplement: Supplementary file 1 [file tg5c00038_si_001.pdf]

Taffit: An Excel Tool for Fitting Tafel Data

Joshua Coduto and Johna Leddy*

Department of Chemistry, University of Iowa, Iowa City, IA 52242, USA

E-mail: joshcoduto@gmail.com

Taffit User Guide and VBA Code

Taffit Sheet Link

The algorithm Taffit is in the Excel® file Taffit.xlsm in the SI. The VBA code is captured below. Also in the SI is an example LSV file. The LSV file GC_LSV_3.TXT is for HER on GC as analyzed in Figure 6 in the paper. The experimental conditions are HER on 0.45 cm^2 GC in H_2 sparged and stirred 0.5 M H_2SO_4, measured without iR compensation at 5 mV s^{-1}. Data were collected on a CH Instruments potentiostat.

Alternatively, the Taffit algorithm is hosted on OneDrive and can be accessed here. Any updates or additions to Taffit will be uploaded to the linked folder, along with any relevant changelogs.

Taffit User Guide

Taffit is built in Excel and requires minimal setup. If not already done, the Solver add-in must be enabled in Excel, and the file Taffit.xlsm must be unblocked by the operating system security to allow running macros.

The developed tool has not been tested with all reaction systems or potentiostat platforms. Development and testing has only used the Windows operating system. Issues, bugs, or feedback should be directed to joshcoduto@gmail.com.

Enabling Solver

Solver is called within the Taffit code and so must be enabled prior to use. To enable Solver in Excel for Windows (Load the Solver Add-in in Excel):

1. Navigate to **File** > **Options** > **Add-ins**.
2. Select **Excel Add-ins** under the **Manage** drop-down and select **Go**.
3. Check the **Solver add-in** box and select **OK**.

Once enabled, Solver should be available in the Data tab under Analyze.

To enable Solver in Excel for Macintosh:

1. Go to **Tools** and select **Add-ins**
2. Under **Add-ins available**, check the **Solver.xlam** box.

Once enabled, the Tools menu should contain the Solver command.

Enabling Macros

Often .xlsm files are marked as security risks by operating systems. The steps needed to enable macros are dependent on the operating system and security settings of the computer. Typically, the user is prompted when opening the file with a security warning. Clicking **Enable Content** will enable macros in the Excel sheet. The user is then prompted to make the file a Trusted Document, which prevents further attempts to disable macros. Alternatively, this may be applicable (Unblock a single file).

If security is of concern, the code provided below can be placed into a new VBA-enabled workbook. The Master Sheet should also be reproduced on the new workbook.

Setting Taffit Parameters

Taffit reads all of system and fitting parameters from the Master Sheet when running. Care should be taken to ensure that all values are set correctly before analysis. Figure 1 shows a sample of the Master Sheet, and Table 1 further details the input parameters and their limitations. As written, the position of inputs is critical and cannot be changed without editing workup macros. For instance, the VBA code always reads the surface area from cell B2.

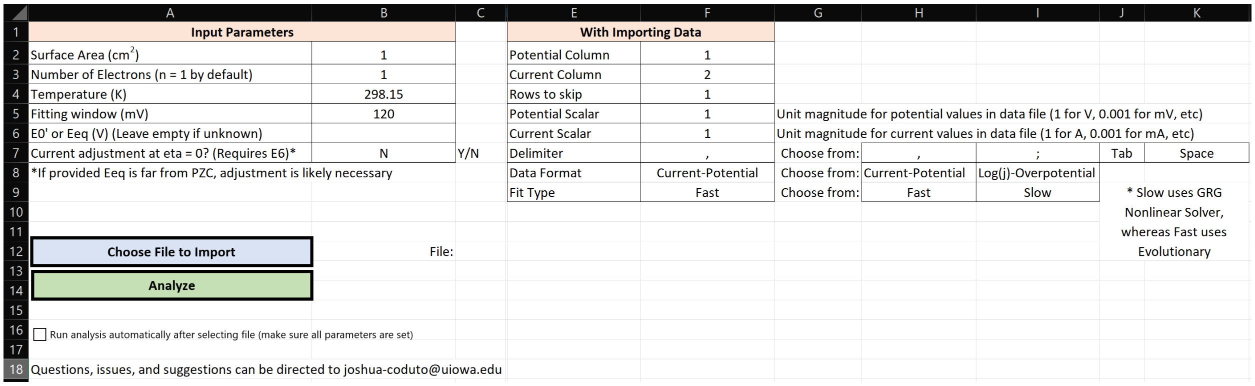

Figure 1: Master Sheet for Taffit operation in Microsoft Excel. Shown are all input parameters and buttons for operation.

Table 1: Input parameters for Taffit Operation

Cell	Parameter	Description	Value restrictions
B2	Surface Area	Surface area of electrode (cm^2). Used to calculate current density. Set to 1 to keep .txt file currents unmodified.	-
B3	Number of electrons	Number of electrons involved in limiting mechanistic step. Assumed to be 1 unless high α values are observed.	Positive integer value
B4	Temperature	System temperature in K	-
B5	Fitting window	Region in mV around $\eta = 0$ in which to fit data. A 120 mV window fits data from +60 to -60 mV.	-
B6	E_{eq} (optional)	Equilibrium potential or OCP, if known. If not provided, automatically calculates E_{eq} as the point of zero charge	Leave empty for auto detection, no other restrictions
B7	Current adjustment (requires E_{eq})	If enabled, automatically subtracts currents at provided E_{eq} to create Tafel form.	Must be either Y or N
F2	Potential column	Column position of potential data file selected for import. Used to read data.	Positive integer value
F3	Current column	As above for current	Positive integer value
F4	Rows to skip	Number of rows containing header information. This is not read when importing.	Positive integer value
F5	Potential scalar	Conversion scale for potential. Potential values are divided by this to convert to V.	-
F6	Current scalar	As F5 for current in amps A	-
F7	Delimiter	Used to separate entries in the data file selected for import	One of four entries in H7:K7
F8	Data format	Used to determine whether data is in E-i or Tafel form. The former is the default option.	One of two entries in H8:I8
F9	Fit type	Used to determine which Solver method to use. **Fast** is recommended but **Slow** is more likely to return a global minimum	One of two entries in H9:I9

File import parameters are set at F2 to F9. File import parameters are specific to different potentiostat manufacturers. Importing parameters need only be set once if using data from a single instrument. It is recommended to use automatic E_{eq} detection by leaving cell B6 empty. If E_{eq} is provided, automatic current adjustment is recommended to offset effects form electrode charging. The "Fast" fit type is recommended for both speed and accuracy. If quality fits are not being found, "Slow" fitting may yield a global σ minimum at the cost

of greater analysis time.

Running Taffit

Once parameters are set, clicking **Choose File to Import** will open a file picker window. The user can select a single .txt or .csv file for import. After closing the dialog box, the file path should be printed in cell C12. If the box in A16 is checked, Taffit will automatically run after a file has been selected. Otherwise, clicking **Analyze** runs the code.

Taffit creates a new sheet in the workbook and names it using the .txt file. Users are prompted before overwriting existing sheets, with the option to rename the new sheet.

After data has been processed, isolated, and simulated, Solver is called to find the conditions of best fit. This process typically takes less than 30 seconds. Throughout the simulation, users may be prompted by the maximum number of iterations, solutions, or time. A global maximum is typically found quickly using Evolutionary fits, so stopping analysis is typically recommended. Solver fitting can always be rerun if fit quality is insufficient.

After completion, the left side of the sheet contains the resulting parameters and graphical representation. The relevant kinetic parameters are highlighted yellow, as is the calculated σ used to assess fit quality. Acceptable fits are generally characterized $\sigma \lesssim 0.1$, but slightly higher σ values may mark adequate fits. In general, a range of η_{win} below mass transport impacts, should yield similar values of the parameters determined by Taffit. If not provided, the automatically detected equilibrium potential is reported in B16.

To change window size after analysis, first change the value in B26. The Fit Type can be changed using the string in B31. Clicking **Re-isolate data and run Solver again** will collect data in the specified potential window and run through analysis steps. If no change in fitting window is needed (such as when changing n), click **Run Solver again**.

Modification is needed if the resulting parameters are equal to the constraints identified in B21:B24. If j_0 is equal to the max or min, adjust the problematic constraint and **Run Solver again**. If α is equal to the max or min, adjustment of n may be necessary.

Common Errors and Debugging

Taffit has not been tested for all potentiostat platforms, instruments, and methods of data recording. Discovery of bugs in operation is anticipated. However, some common points of operational failure have been identified and are detailed below. If errors persist, please contact the manuscript author Josh Coduto (joshcoduto@gmail.com) for assistance.

A "Type Mismatch" error during operation is typically the result of an error in importing data. This is further evidenced when a new sheet is opened but the data are not completely imported before code termination. The program fails when importing lines of data containing text. First, ensure that the "Rows to skip" in F4 and "Delimiter" in F7 are correctly assigned. Next, ensure that no lines in the data file contain non-numeric entries. This is tested by visual inspection of the data file.

If analysis runs to completion but fit quality is poor, it may be necessary to rerun analysis. Check to ensure that the α and j_0 values are not equal to their maxima (as set by the data in the left-hand side of the results sheet). Changing any of these parameters and rerunning analysis may improve results. If $\alpha = 1$, then adjustment of n may be necessary. Changing η_{win} in B5 or changing the Fit Type in F9 may be useful.

There are several exit points when running Solver. Optimization may encounter the maximum number of feasible solutions, maximum run time, or maximum iterations. Users may choose to end optimization at any of these points, as the global minimum has likely been located. However, allowing Solver to continue will not adversely impact results and may identify better optimized conditions. More information on Solver in Excel can be found here.

Visual Basic Code

The following code comprises the Taffit tool. Modification of the code is allowed and encouraged with proper acknowledgement of the original work.

```
Sub FileLocationButton_Click()

    ' Preallocation
    Dim filepath As String, file_name As Variant, auto_taffit As Boolean

    'Check to see if autorun is enabled
    auto_taffit = ThisWorkbook.Sheets("Master Sheet").Range("A15").value

    ' Customize and show the file picker dialog box
    With Application.FileDialog(msoFileDialogFilePicker)
        AllowMultiSelect = False
        .Filters.Add "Text Files", "*.txt;*.csv", 1
        '.Filters.Add "Text Files", "*.txt", 2
        '.Filters.Add "Text Files", "*.csv", 3
        .Show

        ' Check if any items were selected
        If .SelectedItems.Count > 0 Then
            fullpath = .SelectedItems(1)
            Range("C12").value = fullpath

            ' Run the Taffit algorithm if the checkbox is selected
            If auto_taffit = True Then
                TafelFit
            End If
        End If
    End With
```

```
End Sub

Function RenameActiveSheet(newName As String) As String

    ' Pause errors and check if the name already exists
    On Error Resume Next
    ActiveSheet.name = newName
    If Err.Number <> 0 Then
        Dim response As VbMsgBoxResult
        response = MsgBox("A sheet with the name '" & newName & "' already exists. _
        Do you want to overwrite it?", vbQuestion + _
        vbYesNoCancel, "Sheet Name Conflict")

        ' Either delete & replace the file, create a new file, or cancel the function
        Select Case response
            Case vbYes
                Application.DisplayAlerts = False
                Sheets(newName).Delete
                Application.DisplayAlerts = True
                ActiveSheet.name = newName
            Case vbNo
                Dim increment As Integer
                increment = 1
                Do While Not SheetNameAvailable(newName & " (" & increment & ")")
                    increment = increment + 1
                Loop
```

```
                ActiveSheet.name = newName & " (" & increment & ")"
            Case vbCancel
                End
        End Select
        Err.Clear
    End If

    RenameActiveSheet = ActiveSheet.name

End Function

Function SheetNameAvailable(name As String) As Boolean

    'Check whether the sheet name is available
    Dim ws As Worksheet
    On Error Resume Next
    Set ws = ActiveWorkbook.Sheets(name)
    On Error GoTo 0
    If ws Is Nothing Then
        SheetNameAvailable = True
    Else
        SheetNameAvailable = False
    End If

End Function

Function DataImport(fullpath, delimiter, row_skip) As String
```

```
' Convert tab delimiter
If delimiter = "Tab" Then
    delimiter = vbTab
End If

If delimiter = "Space" Then
    delimiter = " "
End If

' Create a new sheet for importing data
Dim newSheet As Worksheet
Set newSheet = ThisWorkbook.Sheets.Add

' Name active sheet after .txt file name
Dim FileName As String
FileName = Mid(fullpath, InStrRev(fullpath, "\") + 1)
FileName = Left(FileName, InStrRev(FileName, ".") - 1)
SheetName = RenameActiveSheet(FileName)

' Read file contents
Dim fileContent As String
Open fullpath For Input As #1
fileContent = Input$(LOF(1), 1)
Close #1

'Split the content into lines
```

```
    Dim lines() As String
    lines = Split(fileContent, vbCrLf)

    ' Loop through lines and import after header lines
    Dim rowIndex As Long
    rowIndex = 1

    For I = row_skip + 1 To UBound(lines)
        Dim values() As String
        values = Split(lines(I), delimiter)

        ' Loop through values and import to the worksheet
        For j = LBound(values) To UBound(values)
            newSheet.Cells(rowIndex + 1, j + 14).value = CDbl(values(j))
        Next j
        rowIndex = rowIndex + 1
    Next I

    DataImport = SheetName

End Function

Sub CreateLabels(ws, potcol, icol, potwin, fit_type)
    ' Format the worksheet with labels
    Range("A1:E38").Interior.ColorIndex = 2

    Cells(1, potcol) = "Raw Potential"
```

```
Cells(1, icol) = "Raw Current"

Cells(1, 6) = "Overpotential (V)"

Cells(1, 7) = "j (A/cm^2)"

Cells(1, 8) = "Log(j) (A/cm^2)"

Cells(1, 10) = "Overpot for Sims (V)"

Cells(1, 11) = "Log(j) for Sims (A/cm^2)"

Cells(1, 12) = "Simulated Log(j) (A/cm^2)"


' Create box for kinetic parameters

Range("A1:B1").Merge False

Cells(1, 1) = "Kinetic Parameters"

Range("A1").HorizontalAlignment = xlCenter

Range("A1").Interior.ColorIndex = 39

Range("A2:B4").Interior.ColorIndex = 36

Cells(2, 1) = "j0 (A/cm^2)"

Cells(3, 1) = "-Log(j0) (A/cm^2)"

Cells(4, 1) = "Alpha"

Cells(5, 1) = "Cathodic Tafel Slope (mV/dec)"

Cells(6, 1) = "Anodic Tafel Slope (mV/dec)"

Range("A1:B6").Borders.LineStyle = xlContinuous


' Create box for system parameters

Range("A11:B11").Merge False

Cells(11, 1) = "System Parameters"

Range("A11").HorizontalAlignment = xlCenter

Range("A11").Interior.ColorIndex = 20

Cells(12, 1) = "Number of Electrons"
```

```
Cells(13, 1) = "Temp (K)"
Cells(14, 1) = "Gas Constant (J/(mol*K)"
Cells(15, 1) = "Faraday's Constant (C/mol)"
Cells(16, 1) = "E0' or Eeq (V)"
Range("A11:B16").Borders.LineStyle = xlContinuous

' Create box for fitting parameters
Range("A20:B20").Merge False
Cells(20, 1) = "Fitting Parameters"
Range("A20").HorizontalAlignment = xlCenter
Range("A20").Interior.ColorIndex = 50
Cells(21, 1) = "Min j0 for fit"
Cells(22, 1) = "Max j0 for fit"
Cells(23, 1) = "Min Alpha for fit"
Cells(24, 1) = "Max Alpha for fit"
Cells(25, 1) = "Error of Estimate"
Range("A25:B25").Interior.ColorIndex = 36
Cells(26, 1) = "Fitting Window (mV)"
Range("A20:B26").Borders.LineStyle = xlContinuous

Range("A30:B30").Merge False
Cells(30, 1) = "Solver Parameters"
Range("A30").Interior.ColorIndex = 22
Range("A30").HorizontalAlignment = xlCenter
Cells(31, 1) = "Fit Type"

'Cells(36, 1) = "Scaling"
```

```
    Columns("A").AutoFit
    Columns("C:O").AutoFit

    Dim targetRange As Range
    Set targetRange = ActiveSheet.Range("B:O")
    targetRange.HorizontalAlignment = xlCenter
    Range("A30:B31").Borders.LineStyle = xlContinuous

    ' Populate initial guesses and known kinetic parameters
    ws.Range("B4").value = 0.5
    ws.Range("B14").value = 8.31446261815324
    ws.Range("B15").value = 96485.3321
    ws.Range("B23").value = 0
    ws.Range("B24").value = 1
    ws.Range("B26").value = potwin

    Cells(31, 2) = fit_type
End Sub

Sub CreateTafelData(ws, potcol, potscale, icol, iscale, eeq, curradj, SA, dataform)
    Dim lastRow As Long
    Dim potRange As Range
    Dim iRange As Range
    Dim potvals As Variant
    Dim ivals As Variant
    Dim logi As Variant
```

```
' Create current and potential variables
lastRow = ws.Cells(ws.Rows.Count, potcol).End(xlUp).Row
Set potRange = ws.Range(ws.Cells(2, potcol), ws.Cells(lastRow, potcol))
Set iRange = ws.Range(ws.Cells(2, icol), ws.Cells(lastRow, icol))
potvals = potRange.value
ivals = iRange.value
logi = ivals


' Adjust potential and current to create Tafel form
If dataform = "Current-Potential" Then

    ' If E0 or Eeq is provided, perform subtraction
    If IsEmpty(eeq) = False Then
        For I = LBound(potvals) To UBound(potvals)
            potvals(I, 1) = (potvals(I, 1) * potscale) - eeq
        Next I

        ' Automatically adjust for current if no value is provided in E7
        If IsEmpty(curradj) = True Then
            curradj = "Y"
        End If

        ' Adjust current at eta = 0 if specified. Otherwise, no change to data
        If curradj = "Y" Then
```

```
        vCritidx = Application.Match(0, potvals, -1)
        icrit = ivals(vCritidx, 1)

        For I = LBound(ivals) To UBound(ivals)
            If I = vCritidx Then
                ivals(I, 1) = 0
                logi(I, 1) = 0
            End If

            If I <> vCritidx Then
                ivals(I, 1) = (ivals(I, 1) - icrit) * iscale / SA
                logi(I, 1) = Log(Abs(ivals(I, 1))) / Log(10)
            End If

        Next I

    Else
        For I = LBound(ivals) To UBound(ivals)
            ivals(I, 1) = ivals(I, 1) * iscale / SA
            logi(I, 1) = Log(Abs(ivals(I, 1))) / Log(10)
        Next I
    End If
End If


' Automatically find eta = 0 by finding minimum of Tafel plot
If IsEmpty(eeq) = True Then
```

```
        For I = LBound(potvals) To UBound(potvals)
            ivals(I, 1) = ivals(I, 1) * iscale / SA
            logi(I, 1) = Log(Abs(ivals(I, 1))) / Log(10)
        Next I

        icrit = Application.Min(logi)
        icritpos = Application.Match(icrit, logi, 0)
        eeq = potvals(icritpos, 1) * potscale

        For I = LBound(potvals) To UBound(potvals)
            potvals(I, 1) = (potvals(I, 1) * potscale) - eeq
        Next I
    End If
End If

' Adjust potential and current to create Tafel form
If dataform = "Log(j)-Overpotential" Then
    For I = LBound(ivals) To UBound(ivals)
        potvals(I, 1) = potvals(I, 1) * potscale
        ivals(I, 1) = 10 ^ (ivals(I, 1)) * iscale / SA
        logi(I, 1) = Log(ivals(I, 1)) / Log(10)
    Next I
End If

' Print values
n = 1
For I = LBound(potvals) To UBound(potvals)
```

```
        If logi(I, 1) <> 0 Then
            ws.Cells(n + 1, 6) = potvals(I, 1)
            ws.Cells(n + 1, 7) = ivals(I, 1)
            ws.Cells(n + 1, 8) = logi(I, 1)
            n = n + 1
        End If
    Next I

    ws.Range("B16") = eeq
End Sub

Sub IsolateAndSimFitData(ws, potwin, nelec, T)
    lastRow = ws.Cells(ws.Rows.Count, 14).End(xlUp).Row
    potwin = potwin / 1000 / 2

    ' Loop through all rows and isolate data within the window
    n = 2
    For I = 2 To lastRow
        eta = Abs(ws.Cells(I, 6))
        If eta <= potwin And eta <> 0 Then
            ws.Cells(n, 10) = ws.Cells(I, 6)
            ws.Cells(n, 11) = ws.Cells(I, 8)
            n = n + 1
        End If
    Next I

    Dim colRange As Range
```

```
Dim minVal As Double
Dim maxVal As Double

' Report the min and max to use for simulation
lastRow = ws.Cells(ws.Rows.Count, 10).End(xlUp).Row
Set colRange = ws.Range("K2:K" & lastRow)
minVal = Application.WorksheetFunction.Min(colRange)
maxVal = Application.WorksheetFunction.Max(colRange)
ws.Range("B21").value = 10 ^ (minVal)
ws.Range("B22").value = 10 ^ (maxVal)
ws.Range("B2").value = 10 ^ ((maxVal + minVal) / 2)
ws.Range("B3").Formula = "=-Log(B2)"
ws.Range("B5").Formula = "=-2.303*B14*B13/(B4*B15)*1000"
ws.Range("B6").Formula = "=2.303*B14*B13/((1-B4)*B15)*1000"


' Record known/initial values in the spreadsheet
ws.Range("B12").value = nelec
ws.Range("B13").value = T

' Simulate data and provide formula for sigma
ws.Range("L2").Formula = "=log(abs($B$2 * (exp(-$B$4 * $B$12 * $B$15 * J2 _
/ ($B$14 * $B$13)) - exp((1-$B$4) * $B$12 * $B$15 * J2 / ($B$14 * $B$13)))))"
ws.Range("L2:L" & lastRow).FillDown
ws.Range("B25").Formula = "=SQRT(SUMXMY2(L2:L" & lastRow & ", _
K2:K" & lastRow & ") / (COUNT(L2:L" & lastRow & ") - 2))"
```

```
End Sub



Sub RunTafelAnalysis(fit_type)
    Application.Calculation = xlCalculationAutomatic
    SolverReset

    ' Perform Evolutionary Solver if chosen
    If fit_type = "Fast" Then
        SolverOK SetCell:=Range("B25"), MaxMinVal:=2, _
        ByChange:=Range("$B$2,$B$4"), Engine:=3
        SolverOptions Derivatives:=1, Convergence:=0.00001, _
        PopulationSize:=250, MaxTime:=60, MutationRate:=0.01, _
        MaxTimeNoImp:=10, Scaling:=True, MaxIntegerSols:=10000
    End If

    ' Perform GRG Nonlinear Solver if chosen
    If fit_type = "Slow" Then
        SolverOK SetCell:=Range("B25"), MaxMinVal:=2, _
        ByChange:=Range("$B$2,$B$4"), Engine:=1
        SolverOptions MultiStart:=True, Derivatives:=1, Convergence:=0.00001, _
        PopulationSize:=250, MaxTime:=60, Estimates:=2, Scaling:=True
    End If

    ' Set optimization parameters for Solver
    SolverAdd CellRef:=Range("B2"), RElation:=3, FormulaText:="=$B$21"
    SolverAdd CellRef:=Range("B2"), RElation:=1, FormulaText:="=$B$22"
```

```
    SolverAdd CellRef:=Range("B4"), RElation:=3, FormulaText:="=$B$23"
    SolverAdd CellRef:=Range("B4"), RElation:=1, FormulaText:="=$B$24"



    SolverFinish KeepFinal:=1
    SolverSolve userfinish:=True
    Application.Calculation = xlCalculationManual
End Sub

Sub PlotFormatting(ws)
    ' Set the x and y data for both traces of the plot
    lastRow = ws.Cells(ws.Rows.Count, 10).End(xlUp).Row
    lastRowRaw = ws.Cells(ws.Rows.Count, 6).End(xlUp).Row
    AlgoY = Range("L2:L" & lastRow)
    AlgoX = Range("J2:J" & lastRow)
    RawX = Range("F2:F" & lastRowRaw)
    RawY = Range("H2:H" & lastRowRaw)

    ' Create a chart around column D
    With ActiveSheet.Columns("D")
        .ColumnWidth = .ColumnWidth * 12
    End With

    Dim Taffit As ChartObject
    Set Taffit = ActiveSheet.ChartObjects.Add(Left:=260, Width:=500, _
    Top:=0, Height:=400)
    ActiveSheet.ChartObjects(1).Activate
```

```
ActiveChart.ChartType = xlXYScatterLines

' Add two series to the plot for simulated and actual data
With ActiveChart.SeriesCollection.NewSeries
    .name = "Experimental Data"
    '.values = Range("H2:H" & lastRowRaw)
    '.XValues = Range("F2:F" & lastRowRaw)
    .values = Range("H2:H10000")
    .XValues = Range("F2:F10000")
    .MarkerStyle = xlNone
End With

With ActiveChart.SeriesCollection.NewSeries
    .name = "Simulated Data"
    '.values = Range("L2:L" & lastRow)
    '.XValues = Range("J2:J" & lastRow)
    .values = Range("L2:L10000")
    .XValues = Range("J2:J10000")
    .MarkerStyle = xlNone
End With

' Change properties of the chart
With ActiveChart
    .PlotArea.Border.Weight = 1.5
    .PlotArea.Border.Color = vbBlack
    .Legend.position = xlLegendPositionBottom
```

```
With .SeriesCollection(2)
    .Format.Line.ForeColor.RGB = RGB(216, 27, 96)
    .Format.Line.Weight = 2
End With

With .SeriesCollection(1)
    .Format.Line.ForeColor.RGB = RGB(30, 136, 229)
    .Format.Line.Weight = 2
End With

' Y axis settings
With .Axes(xlValue, xlPrimary)
    .HasTitle = True
    .AxisTitle.Text = "log(j) (A/cm^2)"
    .AxisTitle.Format.TextFrame2.TextRange.Font. _
    Fill.ForeColor.RGB = RGB(0, 0, 0)
    .MinimumScale = Round(WorksheetFunction.Min(AlgoY) * 1.1, 0)
    .MaximumScale = Round(Range("K2") * 0.9, 0)
    .HasMajorGridlines = False
    .HasMinorGridlines = False
    .Crosses = xlMinimum
    .TickLabels.Font.Size = 16
    .AxisTitle.Font.Size = 18
    .TickLabels.Font.Color = vbBlack
    .MajorTickMark = xlTickMarkCross
    .MinorTickMark = xlTickMarkInside
    .Border.Color = vbBlack
```

```
        .Border.Weight = 1.5
    End With

    ' X axis settings
    With .Axes(xlCategory, xlPrimary)
        .HasTitle = True
        .AxisTitle.Characters.Text = "Overpotential (V)"
        .AxisTitle.Format.TextFrame2.TextRange.Font.Fill.ForeColor.RGB = RGB(0, 0, 0)
        .MaximumScale = Round(Range("J2") * 1.4, 2)
        .MinimumScale = Round(Range("J" & lastRow) * 1.4, 2)
        .HasMajorGridlines = False
        .HasMinorGridlines = False
        .ReversePlotOrder = True
        .Crosses = xlMaximum
        .TickLabels.Font.Size = 16
        .AxisTitle.Font.Size = 18
        .TickLabels.Font.Color = vbBlack
        .MajorTickMark = xlTickMarkCross
        .MinorTickMark = xlTickMarkInside
        .Border.Color = vbBlack
        .Border.Weight = 1.5
    End With

    End With


End Sub
```

```
Sub CreateMacroBox(ws, boxname, boxtext, macroname, boxnum)
    leftPos = 260 + (300 * boxnum)
    topPos = 420
    boxwidth = 200
    boxheight = 30

    ' Create box in set location
    Dim shp As Shape
    Set shp = ws.Shapes.AddShape(msoShapeRectangle, leftPos, _
    topPos, boxwidth, boxheight)

    With shp
        .name = boxname
        .Fill.ForeColor.ObjectThemeColor = 5 + boxnum
        .Line.Weight = 2
        .TextFrame.Characters.Text = boxtext
        .TextFrame.HorizontalAlignment = xlHAlignCenter
        .TextFrame.VerticalAlignment = xlVAlignCenter
    End With

    shp.OnAction = macroname

End Sub
Sub RunTafAgain()
    Set ws = ThisWorkbook.ActiveSheet
```

```
    ' Clear data
    lastRow = ws.Cells(ws.Rows.Count, 10).End(xlUp).Row
    ws.Range("J2:L" & lastRow).ClearContents

    ' Read parameters from cells and run analysis
    potwin = Cells(26, 2)
    n = Cells(12, 2)
    T = Cells(13, 2)
    fit_type = Cells(31, 2)



    IsolateAndSimFitData ws, potwin, n, T
    RunTafelAnalysis fit_type
End Sub

Sub RunFitAgain()
    ' Just run Solver again
    fit_type = Cells(31, 2)
    RunTafelAnalysis fit_type
End Sub

Sub TafelFit()

    ' Read relevant values from the Excel sheet
    delim_input = Range("F7").Text
    SA = Range("B2")
    row_skip = Range("F4")
```

```
potwin = Range("B5")
potcol = Range("F2")
icol = Range("F3")
potscale = Range("F5")
iscale = Range("F6")
n = Range("B3")
T = Range("B4")
dataform = Range("F8").Text
fullpath = Range("C12").Text
fit_type = Range("F9").Text
eeq = Range("B6")
curradj = Range("B7").Text

' Import data and perform preliminary formatting
Dim ws As Worksheet
SheetName = DataImport(fullpath, delim_input, row_skip)
Set ws = ThisWorkbook.Sheets(SheetName)
ws.Activate



potcol = potcol + 13
icol = icol + 13

CreateLabels ws, potcol, icol, potwin, fit_type

' Conduct Tafel analysis
CreateTafelData ws, potcol, potscale, icol, iscale, eeq, curradj, SA, dataform
```

```
IsolateAndSimFitData ws, potwin, n, T
RunTafelAnalysis fit_type
PlotFormatting ws

' Create accessability boxes
boxname = "RunTafBox"
boxtext = "Isolate data and run Solver again"
macroname = "RunTafAgain"
boxnum = 0

CreateMacroBox ws, "RunTafBox", "Re-isolate data and run Solver again", _
"RunTafAgain", 0
CreateMacroBox ws, "RunFitBox", "Run Solver again", "RunFitAgain", 1

End Sub
```
